# Epigenetic control of seed development and dormancy in cereals

**DOI:** 10.1080/15592324.2025.2568929

**Published:** 2025-10-27

**Authors:** Manjit Singh, Karminderbir Kaur, Purnima Kandpal, Zhou Zhou, Wei-Yuan Chen, Jaswinder Singh

**Affiliations:** a Trait Discovery and Optimization, Biotechnology, Corteva Agriscience, Johnston, IA, USA; b Department of Plant Science, McGill University, Macdonald Campus, Lakeshore Road Sainte-Anne-de-Bellevue, Quebec, Canada

**Keywords:** Seed development, seed dormancy, germination, pre-harvest sprouting, epigenetics

## Abstract

Seeds, which are imperative for the propagation of seed plants, are also of major nutritional and economic value in agriculture. The precise dormancy and germination of crop seeds are important traits for modern agriculture. Pre-harvest sprouting (PHS) or the germination of seeds while attached to the plant before harvest, is a significant problem in crops, particularly in cereals. Therefore, understanding the various mechanisms of seed development and dormancy are imperative. While the molecular and hormonal aspects of seed dormancy are well understood, the role of epigenetic pathways is just beginning to unravel, particularly for cereal crops. The majority of this information has been generated in *Arabidopsis*; however, there is increasing focus on cereal crops such as rice and maize. Other important cereal crops, such as wheat and barley, lag behind even though seed dormancy and PHS are even more critical for these crops. Similarly, while much progress has been made in understanding the role of histone modifications in seed development, the role of DNA methylation has not been well investigated. In this article, we review the progress made in uncovering the role of epigenetic modifications in cereal crops with reference to knowledge generated in *Arabidopsis*.

## Introduction

Seed production is a fundamental biological process in the life cycle of seed-bearing plants, as it ensures the continuation of species through the generation of offspring. By acquiring dormancy, seeds have evolved to survive under various adverse conditions, which ensures the survival of the species.^1^^,^^2^ Seeds are also the primary component with nutritional and economic value for major agricultural crops. Cereal crop seeds provide 60% of the calories for human nutrition worldwide, with rice and wheat being the two most consumed cereal crops.^3^^,^^4^ Seed development, therefore, encompasses phases of plant reproductive growth that are deemed essential not only for seed viability but also for seed quality and yield.^5^ Starting from double fertilization, seed development consists of early seed development, early seed maturation and late seed maturation, which includes seed dormancy.^6^^,^^7^ Through double fertilization, the egg cell produces the embryo, and the central cell produces the endosperm.^8^ While the embryo produces the plant for the next generation, the endosperm stores energy reserve and metabolites that support the germination of the seeds.^9^ The endosperm is also the predominant tissue of the cereal grain and has the greatest commercial value. Therefore, understanding seed development is important not only from a biological perspective but also from an agricultural perspective to increase crop yields.

Germination is defined as the emergence of the radicle and hypocotyl from the seeds under the right moisture conditions.^10^ Seed dormancy, a quiescent state of the seed that prevents germination even under favorable conditions, is primarily an adaptive trait of many plant species to weather unfavorable growing conditions. Plant species have evolved various types of seed dormancy mechanisms as a means of survival through the control of germination.^11^ The environmental adjustments caused by seed dormancy are also a key trait for the speciation and diversification of seed-producing plants.^11^ It is believed that the phenomenon of seed dormancy is more prevalent in species that grow in the temperate climate regime to overcome unfavorable growth conditions, such as harsh winters, whereas species growing in tropical conditions do not generally exhibit seed dormancy.^12^ Domestication and subsequent selection and breeding have led to changes in seed characteristics that are more suited for modern agriculture. Seed dormancy is such a characteristic that has been selected against to allow for prompt germination of seeds.

Seeds of many species undergo an after-ripening stage which overcomes the physiological dormancy induced during seed maturation.^12–14^ The seed regulatory network is coordinated by plant hormones of which abscisic acid (ABA) is predominantly involved in making the seeds dormant.^15^^,^^16^ Gibberellic acid (GA) or gibberellins play major roles in germination,^17^ and the ABA–GA balance is an essential feature of the mature seeds.^12^ Thus, the literature converges toward envisaging the development switch between dormancy and germination as represented by the ABA/GAs ratio.^18^^,^^19^

Physiologically mature seeds of many non-dormant cereal varieties can germinate on the plants before harvest under hot and humid weather conditions.^20^^,^^21^ This phenomenon is called pre-harvest sprouting (PHS) and affects many crops, especially cereals such as wheat, barley, maize, and rice, causing annual economic losses of approximately $1 billion worldwide through reduced quality and longevity of the produce.^22–25^ Therefore, resistance to PHS is a highly desirable genetic trait for both public and industrial breeding programs. On the other hand, uniform germination or sprouting is desirable for consistent crop-stand establishment, synchronized maturity and better crop yield.^26^ However, similar to the crucial role of the ABA‒GA balance in seed germination, a delicate equilibrium also exists between resistance to PHS and seed germination.^14^ For many crops, therefore, an important goal is to achieve the right balance of resistance to PHS on the one hand and reduce seed dormancy for rapid and uniform germination on the other.^22^^,^^27^ The rapid germination of seeds is important not only for planting in the field but also for the malting industry.^22^

The major genetic components of seed dormancy, such as genes in the ABA and GA biosynthesis pathways, have been well studied in diverse plant species, including cereals.^28^^,^^29^ Quantitative trait locus (QTL) analysis has identified the seed dormancy-related *Delay of Germination1* (*DOG1*) gene in Arabidopsis,^30^^,^^31^ and the importance of its expression in environment sensing have been well-documented.^32^^,^^33^ Similarly, VIVIPAROUS-1 (VP1) is a major seed dormancy regulator in cereals.^34^ There is increasing evidence that epigenetic modifications play a major role in the regulation of seed dormancy through *DOG1*, *VP1*, and other dormancy‐related genes.^10^^,^^35^ While epigenetics is being explored as an emerging regulation pathway in seed dormancy, the molecular mechanisms mediating these important epigenetic effects are just being revealed, particularly in cereals. In next sections, we focus on several important epigenetic pathways in plants and their association with seed development, dormancy and PHS in the context of cereals.

## Epigenetic pathways in plants

### DNA methylation and the RdDM pathway

DNA, designated as the core of the central dogma, is subject to various regulatory mechanisms before it can express encoded proteins as a trait or phenotype. Epigenetic tagging of RNA and DNA plays a crucial role in determining the course of gene expression and regulation. Epigenetic modifications in plants can occur through (a) DNA methylation, (b) histone modifications and chromatin remodeling, and (c) non-coding RNAs. These three components work synchronously to induce gene expression or repression.

**Figure 1.**
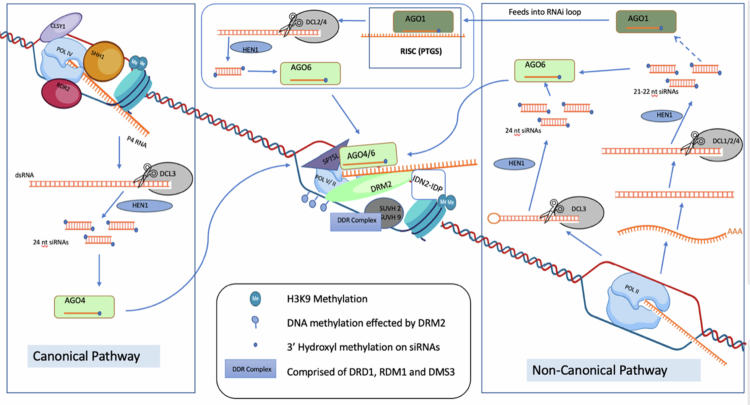
RNA directed DNA methylation pathway. In the conventional RdDM pathway, **Pol IV**-generated transcripts are converted into double-stranded RNAs by **RDR2** and processed into 24-nt siRNAs by **DCL3**. These siRNAs are stabilized by **HEN1** methylation and loaded into **AGO4/6** to form a **RISC complex**. Recruitment of Pol IV is facilitated by the chromatin remodeler **CLASSY1** and the methylated histone readers **SUVH2/9**. On the target side, **Pol V** produces scaffold transcripts that interact with **SPT5L (KTF1)**, which connects the AGO–siRNA complex to the nascent Pol V transcript. Additional co-factors, including **IDN2–IDP complexes** and the **DDR complex (DRD1, DMS3, RDM1)**, stabilize this interaction and help recruit **DRM2** to establish DNA methylation. In contrast, nonconventional RdDM pathways bypass parts of this machinery by using **Pol II-derived transcripts** or other precursors that are processed by **DCL2 and DCL4** into 21–22 nt siRNAs. These siRNAs, often loaded into **AGO2/AGO6**, can guide DNA methylation through partially overlapping mechanisms, sometimes independent of Pol IV/RDR2. Together, both pathways illustrate the versatility of RdDM in integrating multiple siRNA biogenesis routes to reinforce transcriptional silencing.

DNA methylation refers to the addition of a methyl (CH3) group to cytosine in a symmetric (both strands) (CG, CHG; H=A, C or T) or asymmetric (usually hemi-methylated form) (CHH) fashion. In plants, cytosine methylation can occur in various contexts (CG, CHG, and CHH) and is catalyzed by different methyltransferases acting in different DNA methylation pathways.^36–38^ The methylation is executed by either as de-novo methylation directly by DOMAINS REARRANGED METHYLTRANSFERASE (DRM1 and DRM2), homologs of the animal DNA methyltransferases DNMT3,^39^ or indirectly by DECREASE IN DNA METHYLATION 1 (DDM1), a SWI2/SNF2 chromatin remodeler. Maintenance of DNA methylation is predominantly carried out by methylation DNA methyltransferase METHYLTRANSFERASE 1 (MET1) in association with VARIATION IN METHYLATION (VIM),^39^^,^^40^ the chromomethylases CMT2 and CMT3, in association with H3K9me2 proteins^41^ and DRM2.^42^ Both symmetric and asymmetric methylation are highly enriched in heterochromatic transposable elements (TEs) and repeats, leading to transcriptional gene silencing.^43^ The silencing of transcriptional activity is caused by the recruitment of DNA-binding proteins that recognize methylation tags. In certain cases, DNA methylation, when mediated by DNA methyl-readers SU(VAR)3-9 homologs SUVH1 and SUVH3, can activate gene expression.^44^^,^^45^ DNA glycosylases, which include DEMETER (DME), REPRESSOR OF SILENCING1 (ROS1), DEMETER-LIKE2 (DML2), and DEMETER-LIKE3 (DML3), remove 5 mC from DNA.^46^ ROS1 preferentially removes DNA methylation from the 5’ and 3’ ends of genes, thus countering the function of the RNA-directed DNA methylation pathway (RdDM), which is responsible for establishing new methylation.^47^^,^^48^ RdDM (Figure 1) is crucial for various aspects of plant development, including seed development and several other key developmental transitions.^47^^,^^49^ The canonical pathway involves the production of 24-nucleotide siRNAs, initiated through the generation of nascent transcripts by RNA polymerase IV.^50^ The chromatin remodeling factors CLASSY1-4 (CLSY1-4) are required for 24-nt siRNA generation by controlling Pol-IV's chromatin association.^51^ In Arabidopsis, these proteins have both overlapping and unique, locus-specific functions.^51^ RNA Pol-IV produces non-coding RNAs that are further processed into double-stranded RNAs by RNA-dependent RNA polymerases (RDRs).^50^^,^^52^ Subsequent cleavage by DCL3 generates secondary siRNAs that, together with ARGONAUTE proteins, lead to DNA methylation via the action of methyltransferases such as DRM2.^50^^,^^52^ In addition to the canonical RdDM pathway, several small RNA (sRNA) pathways that feed into the canonical pathway exist and are referred to as non-canonical pathway^52^ (Figure 1). Unlike canonical RdDM, the non-canonical pathways usually employ RNA polymerase II and are generally involved in establishing initial DNA methylation at new target loci, such as novel TE insertions, rather than maintaining existing heterochromatin.^52–54^

RdDM is essential for establishing and maintaining DNA methylation patterns that have transgenerational effects on gene expression and phenotype.^47^ The RdDM pathway has also been linked to male and female gametogenesis, genome compaction and reprogramming, and genomic imprinting and gene dosage in plants.^55–61^ Some RdDM factors are themselves imprinted to favor paternal gene expression.^62^ Furthermore, maternally and paternally acting Pol IV elicit different effects on the endosperm. RdDM has been found to interact with histone modifications, such as H3K9 methylation, indicating extensive crosstalk between DNA methylation and histone modifications in plants.^63^ Histone modifications within DNA methylation valleys (DMVs) are involved in regulating seed-specific gene expression, demonstrating the interplay between different epigenetic marks in controlling seed development.^64^ Mutants lacking RdDM components show severe abnormalities in seed production. Thus, the RdDM pathway plays a crucial role during early seed development as discussed further in the section titled ‘Role of Argonaute4/6/9 clade Genes in Reproductive and Seed Development’.

**Figure 2.**
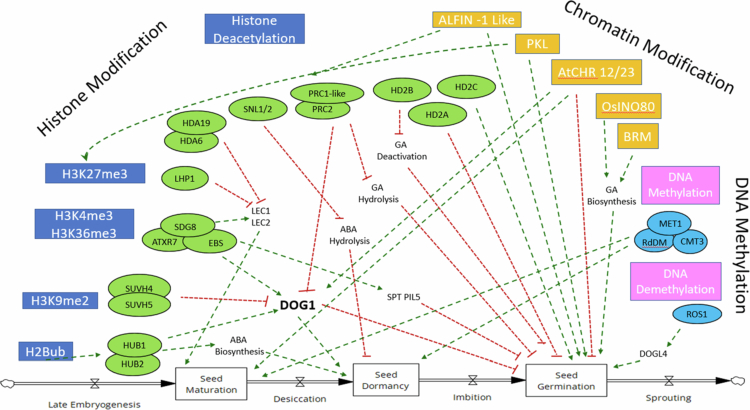
Role of various epigenetic pathways in seed maturation, dormancy and seed germination in *Arabidopsis*: a combination of activating and repressive layers fine-tune DOG1 activity, ensuring proper seed maturation, the establishment of dormancy, and timely germination. Activating pathways include H2B monoubiquitination (H2Bub) by HUB1/HUB2, H3K4me3 deposition by ATXR7, and H3K36me3 mediated by SDG8. The H3K4me2/3 reader EBS reinforces their effect. Remodelers such as BRM and OsINO80 maintain an open chromatin state, while DNA demethylation by ROS1 promotes DOG1 expression. The silencing of DOG1 through repressive pathways allows for seed germination. SUVH4 and SUVH5 deposit H3K9me2, while PRC2 catalyzes H3K27me3. LHP1 binds this mark and recruits PRC1-like proteins, with ALFIN1-like factors and the remodeler PKL further stabilizing the repression. ATCHR12 and ATCHR23 act redundantly with PRC2 to enforce silencing. Histone deacetylases, including HDA6, HDA19, HD2A, HD2B, HD2C, and SNL1/2, compact chromatin and suppress DOG1, partly by modulating ABA signaling. The DNA methyltransferases MET1, CMT3, and the RdDM pathway reinforce silencing, and DOG4L also contributes as a negative regulator.

### Histone modifications

Histones H2A, H2B, H3, and H4 are part of the chromatin around which the DNA molecule is wound.^65^ Covalent modifications of histone proteins can alter chromatin structure, which can make the chromatin accessible or inaccessible to the transcriptional machinery.^66^ Histone modifications generally occur in the *N*-terminal tails of histones and include methylation, acetylation, ubiquitination, and phosphorylation.^66^^,^^67^ Histone methylation, acetylation, and ubiquitination play important roles in plant and seed development (Figure 2, Table 1).

#### Histone methylation

Histone H3 can be methylated at lysine or arginine residues, and depending on the number of methyl groups added at the lysine residue, H3 can be categorized as mono-, di-, or trimethylation, that can have different biological functions. Methylation of the *N*-terminal tail of the histone H3 plays an important role in plant development.^68^ Histone methyltransferases catalyze the addition of methyl groups and vary depending upon the type of methylations. Three partially redundant methyltransferases, i.e., KRYPTONITE/SU(VAR)3–9 HOMOLOGUE 4 (KYP)/SUVH4), SUVH5, and SUVH6 catalyze H3K9 methylation.^69^ H3K27me1 methylation, on the other hand, is mediated by two plant-specific histone methyltransferases, ARABIDOPSIS TRITHORAX-RELATED PROTEIN5 (ATXR5) and ATXR6. Loss of H3K27me1 has been shown to be involved in heterochromatin decondensation and the activation of transposable elements.^70^ Like H3K27me1, the trimethylated state of H3K27 (H3K27me3) executes a repressive role and is preferentially associated with the bodies of inactive genes.^71^ Deposition of H3K27me3 is mediated by the evolutionarily conserved POLYCOMB REPRESSIVE COMPLEX2 (PRC2). PRC genes are essential epigenetic modifiers responsible for maintaining and interpreting histone modifications, thereby creating a heritable chromatin state. In contrast, the removal of histone lysine methylation is catalyzed by Jumonji C domain-containing proteins (JMJs) histone demethylases^72^ (Figure 2, Table 1).

#### Histone acetylation

**Figure 3.**
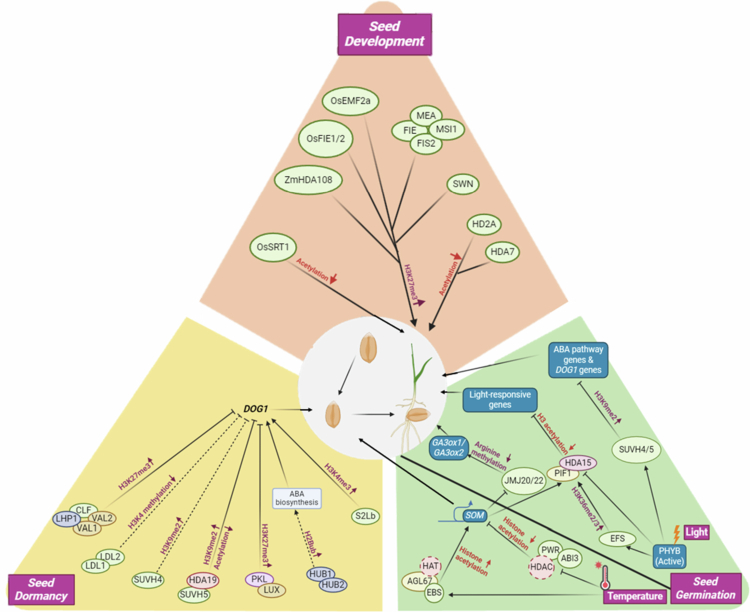
Involvement of various epigenetic factors in seed development, dormancy and germination in cereals. During seed development, histone deacetylases (HDACs), such as OsSRT1, ZmHDA108, HD2A, and HDA7 mediate deacetylation (loss of H3/H4 acetylation) to repress inappropriate transcription. Polycomb group proteins, including OsFIE1/2, OsEMF2a, MEA, FIE, MSI1, FIS2, and SWN, catalyze H3K27me3 through PRC2, ensuring proper endosperm and embryo development. Active marks such as H2B monoubiquitination (H2Bub) by HUB1/HUB2 and H3K4me3 deposition supported by S2Lb promote *DOG1* expression, leading to seed dormancy. Conversely, repressive marks reduce dormancy. SUVH4/5 deposit H3K9me2, CLF and VAL1/VAL2 promote H3K27me3, while LHP1 recognizes this mark and recruits PRC1-like proteins. Histone deacetylases (HDA19, HDAC family) remove acetylation to compact chromatin, while PKL remodels nucleosomes to stabilize repression. LDL1/LDL2 function as H3K4 demethylases, also reducing *DOG1* activity.

Histone acetylation occurs at various lysine residues of H2A, H2B, H3, and H4 histone proteins.^73^^,^^74^ Acetylation of histone lysine residues is a marker of active transcription and opens up the chromatin structure, therefore facilitating transcription; however, deacetylation of histones is associated with transcriptional repression.^75^ The overall dynamics of histone acetylation are controlled by the action of histone acetyltransferases (HATs) and histone deacetylases (HDACs).^74^ During seed development, histone deacetylases (HDACs), such as OsSRT1 (sirtuin-like), ZmHDA108, HD2A, and HDA7, mediate deacetylation (loss of H3/H4 acetylation) to repress inappropriate transcription^76^ (Figure 3, Table 1).

#### Histone ubiquitination

Monoubiquitination is a modification through which a single ubiquitin molecule is covalently conjugated to a lysine residue of a histone protein and is involved in chromatin regulation, and both H2A and H2B can be monoubiquitinated.^77^ Monoubiquitination of histone H2B (H2Bub1) is a marker of active genes and often occurs in combination with H3K4me3 and H3K36me3.^67^ In contrast, H2A monoubiquitination (H2Aub1) is a repressive transcription marker and is closely linked to H3K27me3. In *Arabidopsis*, the E3 ub-protein ligases HISTONE MONOUBIQUITINATION 1 (HUB1) and HUB2 to catalyze H2Bub1 along with the UBIQUITIN CARRIER PROTEIN (UBC1-3) enzymes.^78^^,^^79^ H2Aub1 monoubiquitination is catalyzed by core components of polycomb repressive complex 1 (PRC1), a partially conserved complex that binds H3K27me3.^80^ Deubiquitination is executed in *Arabidopsis* by the UBIQUITIN-SPECIFIC PROTEASE 26 (UBP26/SUP32) enzyme that can remove ubiquitin from H2B.^81^^,^^82^

**Table 1.** Various epigenetic regulators involved in seed development in *Arabidopsis* and in cereals (↓ implies reduction; ↑ implies increase).

S. No.	Epigenetic regulators	Organism	Function	Seed stage	Mark/Process	References
**Histone Deacetylases (HDACs) – epigenetic erasers**						
1	HDA6/HDA7/HDA19	Arabidopsis	Deacetylates H3K9/H3K4; repress *LEC1/2/FUS3*; regulate ABA and light-mediated germination	Germination	H3K9ac/H3K4ac ↓	[83–85]
2	HDA15	Arabidopsis	Works with PIF1 to repress GA20ox1/2 and inhibit germination	Germination (light)	Histone deacetylation	[86]
3	HD2A/HD2B/HD2C	Arabidopsis	Repress *DOG1*; mutants show increased dormancy	Germination	H3K9ac/H3K14ac ↓, H3K9me2	[84],[87]
4	ZmHDA108	Maize	Required for normal development	Development	Histone acetylation ↓	[88]
5	OsSRT1	Rice	NAD^+^-dependent deacetylation; seed/plant development	Development	H3K9ac	[89]
6	SNL1/SNL2	Arabidopsis	Co-repressors; deacetylate ABA/ET genes redundantly	Dormancy	H3K18 deacetylation	[90]
**Histone Methyltransferases – epigenetic writers**						
7	SUVH4 (KYP)/SUVH5	Arabidopsis	Write repressive marks to promote dormancy	Dormancy	H3K9me2 ↑	[83],[91]
8	SDG8 (EFS)	Arabidopsis	Antagonizes PRC2 by evicting it from target genes, promoting transcriptional activation	Development	H3K36me3 ↑	[92]
9	ATXR7	Arabidopsis	Adds methylation at *DOG1, NCED9* to promote dormancy	Dormancy	H3K4me3 ↑	[93]
**Histone Acetyltransferases (HATs) – epigenetic writers**						
10	*OsGW6a*	Rice	Regulates grain weight, hull size, yield, and biomass; higher expression enlarges spikelet hulls and accelerates grain filling	Development	H3/H4 acetylation	[94],[95]
**Histone Readers/Associated Factors**						
11	LHP1	Arabidopsis	Repress *ABI3* and *DOG1* by recruiting PRC1 and later PRC2	Dormancy	H3K27me3 binding	[96],[97]
12	EBS	Arabidopsis	Negatively controls germination under high temperature	Germination (temp.)	H3K4me3 recognition, H4K5ac	[98]
13	ALFIN1-like	Arabidopsis	Repress seed dormancy partly by downregulating *DOG1* expression	Germination	H3K4me3 binding, H3K27me3 switch	[99],[100]
14	S2Lb	Arabidopsis	Knockout triggers reduced seed dormancy and barely detectable *DOG1* expression	Dormancy	H3K4me3	[101]
**Histone Ubiquitination**						
15	HUB1/HUB2	Arabidopsis	Activates *DOG1* and other seed dormancy genes	Dormancy	H2Bub	[93]
**Polycomb Complexes**						
16	PRC1-like	Arabidopsis	H2Aub; repress seed maturation genes	Seedling transition	H2Aub	[97]
17	PRC2 (CLF, SWN, FIE, MSI1, MEA, FIS2, OsFIE1/2, OsEMF2a)	Arabidopsis	Deposit H3K27me3; regulate dormancy, germination, endosperm	Dormancy	H3K27me3	[102]
18	VAL1/VAL2	Arabidopsis	B3-domain TF; binds RY elements in *DOG1, FLC,* and *FT* promoters; recruits PRC2 to repress *DOG1*	Germination	H3K27me3 ↑	[96],[103]
**Chromatin Remodelers (ATP-dependent)**						
19	PKL (PICKLE)	Arabidopsis	Represses *ABI3/ABI5*, thus promoting germination and directly blocks *DOG1*	Germination	H3K27me3↑	[104]
20	AtCHR12/23	Arabidopsis	Stress-responsive chromatin remodelers	Germination	ATP-dependent remodeling	[105]
21	OsINO80	Rice	Knockdown reduces germination, dwarfism	Development/Germination	Chromatin remodeling	[106]
22	BRM	Arabidopsis	SWI/SNF ATPase; regulates seed traits	Dormancy/Germination	Chromatin remodeling	[106]
**Demethylases/DNA Methylation Regulators**						
23	LDL1/LDL2	Arabidopsis	Histone demethylases; flowering/seed transition	Development	H3K4 demethylation	[107]
24	JMJ20/JMJ22	Arabidopsis	Demethylases; repress SOM under light	Germination	H3K9me2 demethylation	[108],[109]
25	ROS1	Arabidopsis	DNA demethylase; derepresses *DOGL4*	Germination	Promoter demethylation	[106]
**Transcription factors/Regulators**						
26	DOG1	Arabidopsis	Master dormancy regulator; dosage sets depth	Dormancy	H3K4me3 ↑ (active), H3K27me3 ↑ (repression)	[110],[111]
27	DOGL4	Arabidopsis	Paralog of *DOG1*; promotes germination and negatively controls dormancy and ABA sensitivity	Germination	DNA methylation	[112]
28	PHYB	Arabidopsis	Photoreceptor TF; degrades PIF1; promotes germination	Germination	Light and temperature signaling	[113]
29	PIF1 (PIL5)	Arabidopsis	Represses germination in dark and high temperature	Dormancy	G-box binding; ABA pathway	[114],[115]
30	SOM	Arabidopsis	Suppresses germination under heat (ABA/GA balance)	Germination (temp.)	Activated by AGL67/EBS, H3/H4ac	[114],[115]
31	AGL67	Arabidopsis	Is MADS-box TF; activates *SOM* with EBS under high temperature	Germination (temperature)	H3K4me3 recognition, H4K5ac ↑	[98]

## Role of epigenetics in seed development and dormancy

In recent years, the importance of epigenetics in seed development has emerged, and various studies have highlighted the crucial role of epigenetic processes such as DNA methylation and histone modifications in this important stage of plant development. As mentioned above, seed development can be divided into three main stages: fertilization and early seed development, early seed maturation (grain filling), and late seed maturation, which includes seed dormancy. The role of epigenetic modifications starts with pre-fertilization development and is active up to the processes of seed dormancy and seed germination.^116–118^

### Role of epigenetics during fertilization and early embryogenesis

While the fertilization of the egg cell produces the zygote, double fertilization of the central cell with a second sperm cell initiates the formation of endosperm.^119^ This process is followed by the coenocyte, cellularization, differentiation, and maturation stages of endosperm development^120^^,^^121^ (Figure 2). The role of methylation in seed development starts prior to fertilization when the central cell undergoes global hypomethylation, primarily due to the expression of *DME* gene.^122^^,^^123^ The sperm cell, on the other hand, remains hypermethylated; therefore, the methylation level in the endosperm is lower than that in the embryo following fertilization. A demethylated maternal genome is consistently found in the endosperm of plants such as Arabidopsis, rice, and maize, indicating that central cell demethylation is a conserved process across species.^124^ Homologs of *DME*, including *ROS1*, *DML2,* and *DML3,* can remove methylated cytosine bases from DNA in any sequence context by means of the base excision repair mechanism and are required for endosperm gene imprinting.^125–128^ Imprinted genes generally promote endosperm development through fertilization independent seed (FIS) class genes of the PRC2 complex, a member of the Polycomb-group (PcG) proteins. The role of these genes is presented in more detail in the next section.

In cereals, genome imprinting and DNA methylation have also been implicated in early seed development in rice and maize. Rodrigues et al. showed that rice endosperm hypomethylation is maternally dependent and occurs mainly within open chromatin regions.^124^ Such demethylation is associated with 24-nt small RNAs derived from both parental genomes and their imprinted genes. Xing et al. reported the dynamic rice embryo and endosperm DNA methylome of the early seed development stage.^129^ Compared to embryo, endosperm hypomethylation primarily occurred in short DNA-TEs, short interspersed transposable elements (SINEs), and non-TE genes. The decreased methylation of DNA-TEs and long terminal repeats (LTRs) implies the distinctive role of demethylation during early endosperm development.^129^ Furthermore, defective rice endosperm caused by interploidy crosses has various epigenetic effects on cellularization during seed development; for example, DNA methylation-mediated endosperm-specific genes are repressed.^130^ The expression of stress-responsive genes was also up-regulated upon the epigenetic changes in the rice endosperm.^130^ Similarly, differentially methylated regions based on parent-of-origin were also identified in maize.^127^ Higgins et al. recently found over 700 imprinted genes were expressed at various stages of endosperm development in eight maize lines, with expression varying across time points.^131^ Interestingly, these authors found evidence for imprinting in three zein genes involved in seed composition, indicating their important role in seed development than previously understood.^131^ The up- and down-regulation of the paternal alleles are mediated by the maternal small interfering RNA pathway by affecting the ratio of the transcripts from the parental genomes in the endosperm.^61^^,^^132^ These sRNA might in turn be regulated through selective imprinting.^58^^,^^61^ Species-specific differences in the origin of these sRNAs have been reported.^124^^,^^133–135^ Thus, DNA methylation represents a dynamic process during endosperm development that affects the expression of genes and siRNAs, thereby affecting seed development. These findings indicate that various important regulators of early seed development are controlled through epigenetic modifications.^136–138^

Following the fusion of the egg and sperm nuclei, a novel pattern of gene expression is activated in the zygote, which includes both paternal and maternal genes, and this process appears to be conserved between dicots and monocots.^139–142^ *MEE1* was the first maternally expressed imprinted gene in the embryo to be identified in maize. The allele-specific expression of *MEE1* correlated with methylation level differences between paternal and maternal alleles and regulated the expression of *MEE1* in the embryo and endosperm.^136^ Although dynamic expression of *MEE1* was detected in early embryos, its function remains unclear.^136^ Studies in *Arabidopsis* have shown that the epigenetic pathways of the maternal lineage control the parental contributions to early embryogenesis.^132^ Raissig et al. found that imprinting in Arabidopsis embryos involves a notable number of genes, suggesting an important role in seed development.^143^ These imprinted transcripts are expressed de-novo following fertilization from either maternal or paternal alleles.^143^ These findings extended the concept of embryo gene imprinting beyond maize and provided evidence that it occurs in dicotyledonous species as well.

## Role of Polycomb Complex Genes (PcGs) in seed development

Endosperm development in Arabidopsis and cereals is regulated by the fertilization-independent seed (FIS) class genes of the PRC2 complex of the Polycomb-group (PcG) proteins (Figure 2). The PRC2-FIS complex genes *MEADEA* (*MEA*), *FIS2*, *Fertilization Independent Endosperm* (*FIE*) and *Multi Suppressor of Ira1* (*MSI1*) prevent endosperm development before fertilization in Arabidopsis, and mutations in these genes result in autonomous endosperm development without fertilization up to the point of cellularization.^144^ *MEADEA* (*FIS1*), *FIS2,* and *FIE* (*FIS3*) genes are imprinted and expressed maternally during endosperm development.^144^^,^^145^ *PHERES1* (*PHE1*) is another gene that has been shown to be paternally expressed and is a master regulator of paternally expressed imprinted genes, as well as of non-imprinted key regulators of endosperm development.^138^ *PHE1* binding sites display different epigenetic modifications on maternal and paternal alleles, which correspond to parent-specific transcriptional activity.^138^ Furthermore, UBIQUITIN-SPECIFIC PROTEASE 26 (UBP26) is necessary for the repression of *PHE1* and thus seed development.^146^ PcG proteins are also required for seed coat initiation following fertilization, further suggesting their role in seed development.^147^

However, in cereals, while many of the PcG genes share sequence homology, they are not completely orthologous to the *Arabidopsis* genes. These genes include the maize homologs PRC2-FIS complex genes *ZmFie1* and *ZmFie2*, both of which exhibit seed-specific expression.^148^ The expression of *ZmFie1* is endosperm-specific and it is imprinted during endosperm development, whereas *ZmFie2* is expressed in the egg cells and more intensively in central cells.^148^ However, knockout mutations of these genes do not produce the autonomous endosperm cell-proliferation phenotype;^149^ therefore, these genes do not mimic the function in *Arabidopsis*. Similarly, in rice, the PRC2-FIS genes, *OsFIE1* and *OsFIE2* are imprinted and expressed during endosperm development^4^^,^^150^ (Figure 2). A mutant of *OsFIE1* did not result in autonomous central cell proliferation in rice,^151^^,^^152^ suggesting that even though *OsFIE1* is imprinted in rice endosperm, it is not involved in the repression of central cell proliferation division prior to fertilization. *OsFIE2* is involved in seed development and grain filling, although it does so with a mechanism distinct from *Arabidopsis.*^4^ A reduction in *OsFIE2* expression led to smaller seeds, partially filled seeds, and partial loss of seed dormancy.^4^ Recently, however, *Osfie2* mutants were shown to display asexual pre-embryo-like structures at a lower frequency without fertilization, and *Osfie1* and *Osfie2* double mutants presented asexual embryo and autonomous endosperm formation at a high frequency.^151^ The *OsFIE2* gene product has been shown to regulate H3K27me3 production in vivo.^4^ *OsEMF2a*, another component of the PRC2 complex, is maternally expressed in the endosperm and is necessary for early seed development.^153^ Delayed cellularization and subsequent autonomous development are observed in the endosperm of the *Osemf2a* mutant.^153^ Similar to the role of *PHE1* in *Arabidopsis,* the loss of *OsEMF2a,* a homolog of *Embryonic Flower2,* reduces H3K27me3 modifications at various type I MADS-box genes involved in endosperm cellularization in rice.^152–154^

## Epigenetics of seed maturation

Following a series of cell divisions and cell differentiation steps, seed development shifts to the maturation phase. The seed maturation can be divided into an early and late maturation phases.^7^ During the early maturation phase, the seed reserves begin to accumulate, and the seeds acquire desiccation tolerance.^5^^,^^7^ This is also called the grain-filling stage and is the most important stage *vis* the economic importance of the cereal grains.^5^ The regulation of many genes involved in the grain-filling stage has been shown to be epigenetically regulated in cereals, although the majority of these studies have focused on the rice.

*OsFIE2* is responsible for the H3K27me3 modification of many endosperm‐specific genes in rice, including *OsMADS6*, a gene with a role in grain filling.^4^ The rice HDAC *OsSRT1* represses the expression of *RICE STARCH REGULATOR1* (*RSR1*) and amylase genes, thus supporting starch accumulation in developing seeds.^89^ Similarly, in maize *hda108* mutants, an increase in acetylated histones H3 and H4 is accompanied by a decrease in H3K9me2, resulting in low fertility in addition to other seed developmental defects.^88^ In rice, the SNF2 helicase *ENDOSPERMLESS1* (*ENL1*) is essential for syncytial endosperm development and plays a role in the maintenance of chromosomal segregation.^155^ *OsLFR*, the rice homolog of the Arabidopsis *LEAF AND FLOWER-RELATED* (*LFR*), is another component that interacts with SWI/SNF complex members and is essential for endosperm development in rice.^156^

CLASSY (CLSY), a family of SNF2 chromatin remodelers, also plays an important role in seed development in rice.^157^ Recently, many imprinted and seed development-associated genes were reported to be regulated through CLSY3-dependent RdDM in rice. *OsCLSY3* is a maternally expressed imprinted gene predominantly expressed in the endosperm, and this expression is regulated by RNA-directed DNA methylation at two tandem transposon elements present in its promoter.^157^ *OsCLSY3* is crucial for endosperm development and grain filling and is a key regulator of endosperm-specific imprinted genes through RdDM.^157^ Castano-Duque et al. identified *CLASSY1* (*CLSY1*) in rice, another gene of the RdDM pathway that enhances anaerobic seed germination.^158^ Interestingly, these authors could not recover homozygous *CLSY1* mutants, suggesting embryonic lethality in the homozygous mutants. Methylation differences were also observed between the *CLSY1* mutants and the wild-type plants under anaerobic germination, particularly at two loci involved in seed development: an E3 ubiquitin-protein ligase and an auxin response factor.^158^ *DME/ROS1* homologs also play a key role in seed development in rice^159^ and maize.^160^ The *ZmROS1a* and *b* genes are involved in regulating gene expression through DNA methylation-sensitive binding of transcription factors, and the *Zmros1ab* double mutant presented reduced seed set and kernel weight.^161^ In rice, the *Osros1a* null mutant produced longer, narrower grains with deformed seeds, characterized by underdeveloped, low-starch endosperms and irregular embryos.^162^

In barley, a series of methylation pathway enzymes have been analyzed, and their expression has been profiled to assign putative functional roles in various stages of endosperm development.^163^ During the prestorage phase of seed development, the high activity of *HvMET1* and *HvCMT1* possibly maintains DNA replication and suppresses seed-specific maturation pathways, whereas the expression of the methyltransferases *HvDNMT3-2* and *HvCMT2* suggested a role in starch accumulation. DEMETER (*HvDME*) serves as an epigenetic regulator owing to its differential expression and methylation in the gene body between two distinct barley cultivars.^164^ Dai et al. analyzed the expression of the DNA methyltransferase genes *TaMET1, TaMET2a, TaMET2b, TaCMT,* and *TaMET3* in wheat and showed that they were expressed in developing seeds, with *TaMET1* and *TaMET3* showing elevated expression in dry seeds.^165^

## Role of epigenetic modifications in seed dormancy

The major genetic components of seed dormancy, i.e., the genes of the ABA and GA biosynthesis pathways, have been well studied in diverse plant species, including cereals.^28^ ABA is the core hormone involved in the maintenance of seed dormancy and the inhibition of seed germination.^16^^,^^29^ Mutant and biochemical studies have been important for dissecting the role of ABA biosynthetic genes in seed development, with recent work identifying additional signaling components^166–169^ (Figure 3).

DOG1 is a key dormancy regulator in Arabidopsis, with *dog1* mutants being completely nondormant.^30^ The DOG1 protein is abundant in freshly harvested seeds but is modified during after-ripening, which likely renders it nonfunctional.^170^ It has been proposed that DOG1 acts as a timer for seed dormancy release, functioning independently but likely in parallel to ABA.^170^ Seed germination is repressed through H3K4me3 and H3K36me3 deposition mediated by ARABIDOPSIS TRITHORAX-RELATED7 (ATXR7) and SET DOMAIN GROUP 8 (SDG8), respectively, both of which activate *DOG1.*^171^ The H3K4me2/3 reader EARLY BOLTING IN SHORT DAY (EBS) reinforces their effect.^172^ The PP2C phosphatases AHG1, AHG3, and RDO5 also interact with DOG1.^173^ Genetic evidence shows that AHG1 and AHG3 function downstream of DOG1 and are crucial to its activity. These findings support the idea that DOG1 maintains seed dormancy by inhibiting these ABA-interacting PP2C phosphatases, which integrate signals from both the ABA pathway and the DOG1 pathways.^173^ Several histone-methylation mediated repressive marks silence *DOG1* to allow germination. The B3 domain-containing transcriptional repressors VIVIPAROUS1/ABI3-LIKE1/HIGH‐LEVEL EXPRESSION OF SUGAR INDUCIBLE2 (HSI2/VAL1) and HSI2‐LIKE1 (HSL1)/VAL2 recruit Polycomb components to deposit H3K27me3 at the RY elements of the *DOG1* promoter.^174^ The chromatin remodeling factor PICKLE (PKL) also plays a role in seed development, and mutants with reduced PKL activity exhibit increased seed dormancy.^175^^,^^176^ It has been shown that PICKLE controls seed dormancy through direct repression of *DOG1.*^176^ LUX ARRHYTHMO (LUX), a member of the evening complex (EC) of the circadian clock, physically interacts with PKL and recruits it to the chromatin region of *DOG1*. Levels of H3K27me3, a repressive mark at specific *DOG1* chromatin loci, are greatly reduced in the *lux* and *pkl* mutants, increasing dormancy compared with the wild type.^176^ REF6 demethylates H3K27me3, increasing ABA breakdown and reducing dormancy by upregulating CYP707A1/3.^174^ In Arabidopsis seeds, ROS1 is active and promotes the expression of the imprinted gene *DOGL4* in both the endosperm and embryo.^112^ ROS1 is present in dry seeds, and certain regions rely on its activity for normal methylation levels.^177^ This balance between active and repressive marks creates a dynamic chromatin environment that allows seeds to switch between quiescent and germinating states.

Histone H2B monoubiquitination HUB1/RDO4 and the histone methyltransferase KYP/SUVH4 also play a key role in seed dormancy.^35^^,^^178^ The *hub1/rdo4* mutant was initially identified as a reduced dormancy mutant along with *hub2,* both of which are required for histone H2B monoubiquitination.^178^ The role of KRYPTONITE (KYP/SUVH4) in seed dormancy was discovered after the initial discovery of this mutant.^69^ Mutations in this gene caused increased seed dormancy, whereas overexpression of this gene reduced seed dormancy in *Arabidopsis*. Several seed dormancy-related genes were up-regulated in the *kryptonite-2* (*kyp-2*) mutant, including *DOG1* and *HUB1.*^35^

DOG1 belongs to a small protein family that has been shown to be conserved in other plant species. *DOG1* homologs have been identified in several cereal species, including rice,^179^^,^^180^ wheat and barley.^181^ In rice, a previously identified high effect seed dormancy QTL, *qSd-1-1*, was shown to encode a DOG1-like protein named OsDOG1L-3. The expression of *OsDOG1L-3* was positively correlated with seed dormancy and was induced by ABA.^180^ Although the sequence conservation of the wheat and barley homologs of *DOG1* was low, they exhibited functional conservation with DOG1. The ectopic expression of wheat and barley *DOG1*-like genes induces seed dormancy in *Arabidopsis.*^181^

HDACs have been found to be key players during early seed development in different plant species; however, limited evidence has indicated that HATs are functionally related to this process.^182^ HDA6 and HDA19, members of the HDAC family, redundantly silence embryonic genes after germination in Arabidopsis.^183^ HDA6 also deacetylates factors involved in ABA signaling, inhibiting downstream gene transcription.^184^ HDA19 facilitates the transition from seed development to germination by interacting with HIGH-LEVEL EXPRESSION OF SUGAR-INDUCIBLE GENE2-LIKE 1 (HSL1) to suppress seed maturation genes.^184^ HD2B histone deacetylase has also been identified as a genetic factor associated with seed dormancy in Arabidopsis. It is known to mitigate dormancy possibly through the repression of negative regulators of GA biosynthesis genes.^185^ HDA6 and HDA19 also associate with HDC1 to promote histone deacetylation and modulate ABA pathway genes, enhancing germination.^186^ The Arabidopsis SWI-INDEPENDENT3 (SIN3)-LIKE proteins SNL1 and SNL2 also decrease histone acetylation at genes central to the ethylene and ABA pathways.^187^

Like DOG1*,* VIVIPAROUS1 (VP1) is a major regulator of seed development in cereals.^34^ *Vp1,* first discovered in maize, and its *Arabidopsis* homolog, *ABA INSENSITIVE3* (*ABAI3*), encode a transcription factor containing A1, B1, B2, and B3 domains.^188^^,^^189^ VP1 has a dual role in the activation of embryo development genes and the repression of germination genes during seed development in maize.^190^ The *vp1* mutants lack anthocyanin due to failure of seed maturation, leading to viviparous embryo development.^190^ Similar to those in maize, mutations in the *OsVP1* gene resulted in severe PHS compared with the wild-type NIP genotype, with seed germination rates of 77.7% and 3.0%, respectively.^75^ OsVP1 also interacts with the TRAB1 transcription factor, which in turn binds to ABA-responsive elements (ABREs) to affect ABA-regulated transcription.^191^ The expression of *OsVP1* is in turn controlled by the rice PcG gene *OsEMF2b.*^192^ ABA treatment of germinating maize seeds induces *VP1* gene expression through selective histone acetylation of its promoter.^193^

In addition to histone modifications, it has been observed that dynamic DNA methylation changes occur during seed development in several species.^87^^,^^129^^,^^177^^,^^194–197^ In Arabidopsis, a decrease of DNA methylation in the mutants *Decrease of DNA Methylation 1* (*DDM1*) and *Methyltransferase 1* (*MET1*) impact seed development.^198^ In *Arabidopsis*, CHH hypermethylation within transposable elements (TEs) is both RdDM and CMT2 dependent during seed development.^177^ To further elucidate the function of hypomethylated regions, Chen et al. discovered that DNA methylation valleys (DMVs) in Arabidopsis and soybean chromosomes are vital for seed development.^199^ These regions show dynamic histone modifications and contain many transcription factors and developmental genes with expression patterns that differ by seed stage, region, and tissue.^199^ Through transcriptional analysis, DNA methylation has also been implicated in GA-induced programmed cell death (PCD) in the aleurone of germinating maize seeds.^150^ The inhibition of DNA methylation also inhibited PCD in maize seeds by regulating reactive oxygen species (ROS)-related gene expression and thus inhibiting the GA-induced production of hydrogen peroxide (H_2_O_2_).^150^ This finding suggests a direct role of DNA methylation in the PCD of the cells of the aleurone layer and therefore in seed germination.

## Role of argonaute4/6/9 clade genes in reproduction and seed development

Argonaute (AGO) proteins are the key players in RNA interference in eukaryotes. They associate with small RNAs (sRNAs) to mediate the transcriptional or post-transcriptional silencing of target genes, thereby regulating diverse biological processes.^200^ In eukaryotes, AGOs generally contain four domains – the *N* domain, Piwi Argonaute Zwille (PAZ) domain, middle (MID) domain, and *P* element-induced wimpy testis (PIWI) domain.^200^^,^^201^ Each AGO domain has a specific function essential to the various physiological roles of AGO proteins: (i) the *N*-terminal domain of the Argonaute protein includes a variable *N*-terminal region that serves as a wedge to slice duplexes during RISC assembly;^202^ (ii) the PAZ domain anchors the 3’ end of the bound small RNA;^203^ (iii) the MID domain, along with the (iv) PIWI domain, is present at the C-terminus and serves as a binding pocket at the junction so that the 5’ end of the small RNA can anchor.^203^ Together, these domains work to correctly position the small RNA sequence relative to the RNA target.

**Table 2.** Role of AGOs in various aspects of plant development.

Function	AGO name	Organism studied	References
**Biotic stresses resistance**			
Aphid infestation	AGO5	Wheat	[204]
Rice dwarf virus (RDV) and rice strip virus (RSV)	AGO1 and AGO18	Rice	[205],[206]
Rice black steak dwarf virus	AGO2	Rice	[207]
Potato virus X, tomato ringspot virus, tobamovirus, and tombusvirus (TCV)	AGO2	Nicotiana benthamiana	[208–210]
Leaf stripe of barley	AGO1, AGO2, and AGO4	Barley	[211]
**Abiotic stress resistance**			
Drought stress	AGO1	*Arabidopsis*	[212]
Drought stress	AGO1, 2b, 4, 5, 7, 9, 10b, and 18a-b	Maize	[213]
Salinity stress	AGO2, 5, 6, 9 and 17	Wheat	[214]
**RNA dependent DNA methylation**			
Binds with miRNA and the complex affects gene transcription	AGO1	*Arabidopsis*	[215]
Chromatin modification	AGO4	*Arabidopsis*	[216]
DNA methylation	AGO4 and AGO6	*Arabidopsis*	[217]
DNA methylation	AGO4a and 4b	Rice	[218]
**Plant development**			
Gametophyte development	AGO4a, AGO5b, AGO6, and AGO9	Wheat	[214]
Gametophyte development	AGO8	*Arabidopsis*	[219]
Anther development	AGO2	Rice	[220]
Leaf development	AGO1 and AGO7	*Arabidopsis*	[221],[222]
Leaf development	AGO7	Maize	[223]
Induction of male sterility	AGO18	Rice	[224]
Reproductive development	AGO17	Rice	[225]
Reproductive development	AGO5 and AGO9	*Arabidopsis*	[226]

The number of ARGONAUTE proteins varies across different species due to the diversification of RNA interference (RNAi) pathways; for example, there are 10 *ARGONAUTE* genes in *Arabidopsis,*^200^ 19 in rice, 17 in maize^227^ and 69 in wheat.^228^ These AGOs are generally divided into 3 major clades according to their phylogenetic relationships: AGO1/5/10, AGO2/3/7, and AGO4/6/8/9.^229^ All these AGOs play different roles in plant development, ranging from pathogen defense to DNA repair to the morphological development of plants (Table 2). Despite encoding diverse proteins that have distinct expression patterns, both spatially and temporally, genes of the AGO4/6/9 clade are partially redundant in their function in the RdDM pathway.^230–232^ This can likely be attributed to the evolutionary divergence of the AGO proteins, which have undergone extensive gene duplication and neofunctionalization.^233^

**Figure 4.**
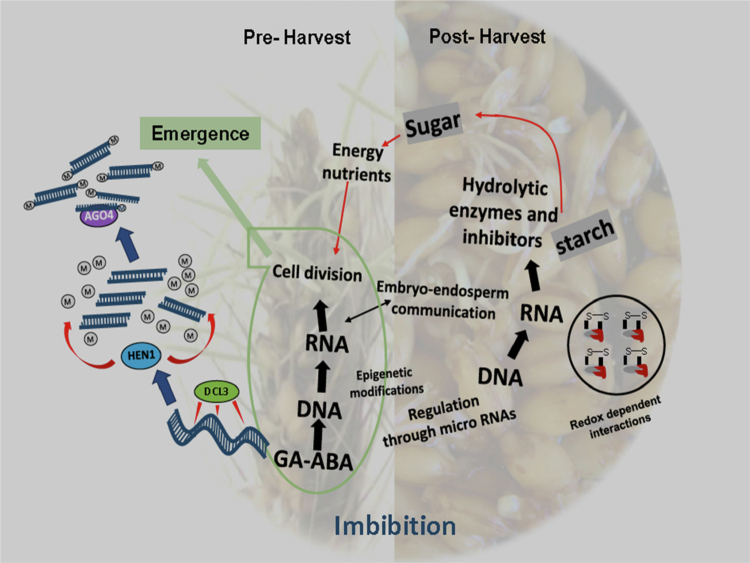
Role of the RdDM pathway in the seed germination process. During pre-harvest imbibition due to rainfall, the RdDM pathway (Pol IV, RDR2, DCL3, and AGO4) and HEN1-stabilized siRNAs, together with specific microRNAs, maintain dormancy by reinforcing ABA signaling, repressing GA-responsive genes, and limiting embryo–endosperm communication. This control extends to starch degradation and sugar transport, restricting the metabolic energy required for premature germination. In contrast, during post-harvest imbibition, reduced RdDM activity and shifts in microRNA profiles promote GA signaling, activate starch and sugar metabolism, and release the repression of DNA replication and cell cycle progression. These changes are accompanied by redox-dependent reactions, including ROS accumulation and antioxidant responses, which act as signaling cues to stimulate germination and seedling establishment.

The importance of small RNAs, DNA methylation and the RdDM pathway in plant reproduction is still being discovered.^234^ Several studies in the past decade have revealed the important role of ARGONAUTES in reproductive development, particularly in pre-fertilization stages of sporogenesis and gametogenesis^229^^,^^231^^,^^232^^,^^235–237^ (Figure 4). Members of the AGO4/6/9 clade of ARGONAUTES play critical roles in reproductive development and are effectors in the silencing of transposons and heterochromatin through 24 nt siRNA.^71^ Even in instances where the ARGONAUTES are implicated in very specific developmental pathways, an underlying function of the suppression of transposons, repeats and heterochromatin through DNA methylation is always present.^231^^,^^232^^,^^238^ AGO4 in *Arabidopsis* and its homologs in other species are known to direct DNA methylation through the RNA-dependent DNA methylation (RdDM) pathway.^232^^,^^239^^,^^240^ The sRNA pathway has been shown to play important role in both seed development and seed germination. Coordinated targeting of DNA methylation sites during seed development, involving RdDM and other methylases, regulates transposable elements crucial for seed germination and development.^241^

**Figure 5.**
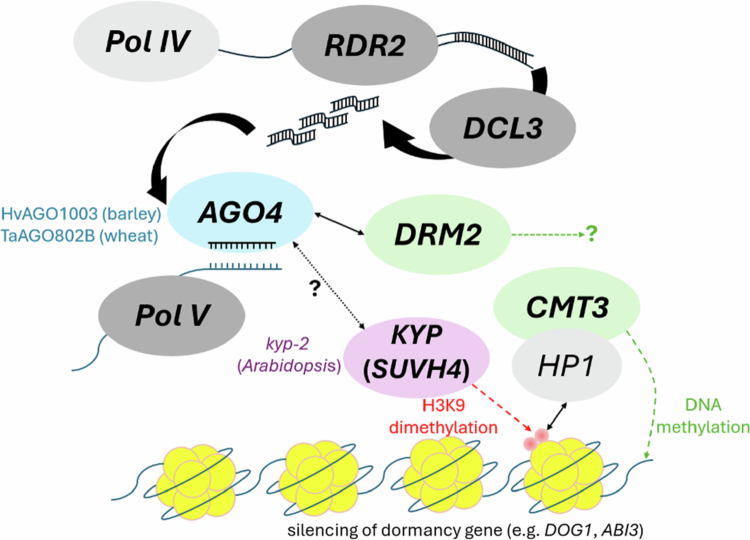
Role of the RdDM pathway in seed dormancy and germination. The RNA-directed DNA methylation (RdDM) pathway establishes transcriptional silencing at key dormancy-associated loci, such as DELAY OF GERMINATION1 (DOG1) and ABSCISIC ACID INSENSITIVE3 (ABI3). In this pathway, RNA Polymerase IV (Pol IV) initiates the production of single-stranded RNAs, which are converted into double-stranded RNAs by RNA-DEPENDENT RNA POLYMERASE 2 (RDR2). These are processed into 24-nt small interfering RNAs (siRNAs) by DICER-LIKE 3 (DCL3) and then loaded onto ARGONAUTE 4 (AGO4). Guided by siRNAs, AGO4 complexes recruit the methylation machinery to homologous genomic regions. The DNA methyltransferase CHROMOMETHYLASE 3 (CMT3) and the histone methyltransferase KRYPTONITE (KYP) reinforce silencing through the deposition of CHG methylation and histone H3 lysine 9 dimethylation (H3K9me2), respectively. The resulting heterochromatic state stably represses the transcription of DOG1 and ABI3, contributing to the regulation of seed dormancy and germination timing.

The RdDM pathway, is particularly active during early seed development, with mutants lacking RdDM components exhibiting severe seed production defects.^242^^,^^243^ In *Brassica rapa*, the loss of RdDM has been associated with seed abortion.^49^ The maintenance of DNA methylation patterns during seed germination, particularly dynamic CHH methylation, is regulated by the RdDM pathway.^49^ Recent studies, however, point towards a role for DNA methylation in seed dormancy, particularly through the RdDM pathway (Figure 5). Palomar et al. demonstrated the involvement of the AGO4/RdDM pathway in germination under salinity stress, revealing differential accumulation levels of transcripts related to dormancy and germination in RdDM mutants compared to wild-type plants in Arabidopsis.^244^ Genes related to abscisic acid (ABA) signaling and metabolism in Arabidopsis, which are essential for maintaining seed dormancy, are regulated through the RdDM-pathway.^245^ This epigenetic regulation ensures that seeds remain dormant under unfavorable conditions, preventing premature germination and ensuring that seedling emergence occurs under optimal circumstances. The *AGO1003* gene of the AGO4/6/8/9 clade in barley is highly expressed in the embryos of developing seeds in barley.^246^ Interestingly, this gene exhibited differential expression in the embryos of dormant and non-dormant varieties, with higher expression levels in PHS-susceptible varieties.^246^

Another study in wheat highlighted the expression of *AGO802B*, a wheat ortholog of the AGO4_9 gene, during grain development (5–20 d after pollination). While all three *AGO802* homeologs were expressed throughout seed development, the expression of *AGO802B* was highest at 20 and 25 d after pollination (DAP). Furthermore, *AGO802B* expression is significantly lower in PHS-resistant varieties compared to susceptible varieties.^247^ A 169bp short interspersed nuclear element (SINE) insertion in the 3’ untranslated region (3’UTR) of *AGO802B* is predominantly found in PHS-resistant wheat varieties, suggesting that *AGO802B* functions as a negative regulator of dormancy.^247^ Although it is not yet clear whether specific coding genes are silenced via RdDM in wheat seeds, analysis of 5S ribosomal DNA from PHS-resistant and PHS-susceptible varieties revealed reduced methylation in PHS-resistant varieties.^247^ Subsequent investigations revealed that the presence of SINE insertions in *AGO802B* led to a 54.7% lower methylation rate.^248^ This finding supports the hypothesis that *AGO4* wheat homologs enhance both histone and DNA methylation, thereby acting as a negative regulator of seed dormancy.^249^ The potential involvement of AGO4 in regulating seed dormancy has been suggested through studies on cereal seed dormancy.^250^

Another study to identify PHS-related QTLs in spring wheat, Dhariwal et al. identified several QTLs that map to known PHS-resistance genes, but also discovered two new QTLs that are located with known epigenetic factors with prior implications in seed dormancy, including *HUB1* and homeologs of the *AGO802* gene, *AGO802A* and *AGO802D.*^21^ Interestingly, the *AGO802-B* homeologs was not identified as a major effect QTL in this mapping population, which suggested some background variation with respect to the effect of this gene.

## Current perspectives and future directions

Seed dormancy is an important agronomic trait, both because of the losses due to preharvest sprouting and the need for prompt germination of seeds at the optimal time.^18^^,^^251^ Much progress has been made in understanding the mechanisms of seed development and germination, and the underlying transcriptional networks have been well studied.^30^^,^^170^^,^^174^^,^^176^ Despite remarkable progress in understanding seed epigenetics, the field still faces substantial gaps and exciting opportunities. Although the role of epigenetics in early seed development is relatively well understood, its role in late seed development and seed maturation needs to be explored further. Understanding the many facets of histone methylation and acetylation/deacetylation in concert with the complete spectrum of DNA methylation to establish specific chromatin states in seed development will be important.

In general, the epigenetic regulation of major seed traits between *Arabidopsis* and cereals, particularly rice, shows overlap but also points towards divergence. This highlights the need for further investigation of the complex network of epigenetic regulation influencing seed development in cereals. Cross-species comparative studies remain underutilized; most mechanistic knowledge comes from Arabidopsis. Expanding research to cereals such as rice, wheat, and barley will reveal conserved versus species-specific epigenetic pathways and evolutionary pressures shaping seed epigenomes. In addition to classical DNA methylation and histone modifications, emerging evidence highlights the roles of non-coding RNAs including lncRNAs, circRNAs and phased siRNAs, and RNA modifications such as m6A, in orchestrating embryo, endosperm, and seed coat development.^123^^,^^198^^,^^252^ Investigating the crosstalk between DNA methylation, histone marks, and RNA-mediated regulation in seed-specific contexts remains a critical frontier.

Multi-omics approaches, ideally in a time-series spanning from embryogenesis through germination, will help link specific epigenetic modifications to physiological outcomes. Single-cell and spatial epigenomics can enable unprecedented resolution of these processes, revealing cell type-specific chromatin landscapes that were previously marred by bulk tissue analyses.^253^^,^^254^ Future work integrating single-cell ATAC-seq, ChIP-seq, and methylome mapping with spatial analyses could uncover heterogeneity across the embryo and endosperm. These tools would also be useful for predicting the developmental trajectories of distinct cell populations within the seed. The massive amount of epigenomic data thus generated can be harnessed to identify potential biomarkers in crops. Machine learning algorithms and AI models will be crucial for integrating a bird’s-eye view of genes, gene expression, and their epigenetic regulation, although these predictions will require validation under laboratory and field conditions. The emerging techniques for epigenome editing can allow direct study of the function of precise epigenetic modifications.^255^ This, coupled with high-efficiency gene editing protocols already available in cereals, would be key to effective epigenetic engineering, particularly for polyploid crops.^256^^,^^257^ Epigenetic engineering seems promising for delving deeper into the epigenetic states of seed development. This would offer opportunities for crop improvement and the exploitation of natural epialleles to optimize seed traits such as dormancy, stress resilience, and nutrient content.^258^^,^^259^

Seed development is highly plastic and sensitive to environmental cues. Understanding how maternal conditions, nutrient availability and environmental cues shape epigenetic marks and whether these changes leave an epigenetic “memory” affecting dormancy and germination will also be of interest.^260^^,^^261^ However, long-term and transgenerational epigenetic effects remain poorly understood. Future work should investigate how parental environments influence seed epigenomes. In this review, we identified several gene targets associated with epigenetic phenomena underlying seed dormancy and germination. It will be interesting to explore how the epigenomic landscape changes between the stages of seed development and seed maturation and prior to germination. This could help identify ways to address problems associated with seed germination. PHS is one such trait of current importance given the imminent and unpredictable climate change. Further studies will help decipher whether sprouting and germination are related to similar pathways or whether there non-hormonal pathways at the epigenetic level, delineating the two processes? Breeders need to develop PHS-tolerant varieties while maintaining the balance between germination and sprouting. Understanding the epigenetic landscape of the genome will be key to unraveling the vital micro-developmental stages before and during germination when epigenetic marks are reset. This could in turn be the foundation to truly sprouting tolerant cereal cultivars.

Box 1. **Epialleles** – Variants of a gene that have identical DNA sequences but differ in their epigenetic marks, leading to differences in gene expression.

**Imprinting** – An epigenetic phenomenon where only one allele of a gene (either maternal or paternal) is expressed while the other is silenced, usually via DNA methylation or histone modifications.

**Gene silencing** – The heritable or stable repression of gene expression achieved through epigenetic mechanisms, including promoter DNA methylation, repressive histone modifications, chromatin compaction, or RNA-mediated pathways such as siRNA-directed silencing.

**Transcriptional activation** – The process by which chromatin modifications, transcription factors, and coactivators create a permissive chromatin environment facilitating the recruitment of RNA polymerase II and thereby increased mRNA production.

**Transcriptional repression** – The process by which genes are maintained in a transcriptionally inactive state through recruitment of repressor complexes, deposition of repressive histone marks DNA methylation, or chromatin remodeling that limits accessibility of the transcriptional machinery.

**RdDM pathway (RNA-directed DNA methylation)** – A plant-specific epigenetic pathway in which small RNAs guide the methylation of DNA at specific loci, leading to transcriptional gene silencing, particularly of transposons and repetitive sequences.

**miRNAs (microRNAs)** – Endogeneously encoded, small (~21–24 nucleotides) non-coding RNAs that regulate post-transcriptional gene expression by binding to target mRNAs, resulting in mRNA degradation or translational repression.

**siRNAs (small interfering RNAs)** – Double-stranded, 21–24 nucleotide RNAs derived from exogenous or endogenous sources that mediate sequence-specific RNA interference, directing either mRNA degradation or epigenetic modifications such as DNA methylation via the RdDM pathway.

**PcG proteins (Polycomb group proteins)** – Conserved multiprotein complexes that mediate long-term transcriptional repression by catalyzing histone modifications such as H3K27me3 and establishing compacted chromatin states, thereby regulating developmental gene expression and cellular identity.

**Chromatin remodeling** – The dynamic modification of chromatin structure by ATP-dependent complexes (e.g., SWI/SNF, ISWI, CHD), allowing or restricting access of transcription machinery to DNA and thereby regulating gene expression.
